# Determinants of obstetric fistula in Ethiopia

**DOI:** 10.4314/ahs.v17i3.9

**Published:** 2017-09

**Authors:** Asrat Atsedeweyn Andargie, Abebe Debu

**Affiliations:** 1 Department of Epidemiology and Biostatistics, University of Gondar, Ethiopia; 2 Department of Statistics, Jimma University, Ethiopia

**Keywords:** Obstetric fistula, logistic regression, determinant factors

## Abstract

**Background:**

Obstetric fistula is a maternal morbidity creating devastating health problems for the women. Continuous and uncontrollable leaking of urine or faeces from vagina can lead to life changing stigmatization for women in third world countries. The underlying factors and consequences of this problem are not yet fully identified and adequately documented in Ethiopia.

**Methods:**

This study is based on the Ethiopian Demographic and Health Survey data (EDHS, 2005). The survey collected information on a total of 14,070 women who were interviewed face to face on their background characteristics as well as reproductive health issues, out of which 3178 women had complete measurements and were considered in this study. Descriptive and binary logistic regressions techniques were used using demographic, socio-economic, health and environmental related variables as explanatory variables and status of obstetric fistula as a response variable.

**Results:**

The results showed that geographical region, place of residence, educational status, age at first birth, age at first marriage, employment status, place of delivery and follow up of antenatal care during pregnancy were significant determinant factors of obstetric fistula in Ethiopia.

**Conclusion:**

The study showed that demographic, socio-economic, environmental and health related variables have an import ant effect on determinants of obstetric fistula in Ethiopia.

## Introduction

Obstetric fistula is a child birth injury usually caused by unrelieved, prolonged obstructed labor. Obstructed labor can develop during the second stage of labor, when the fetus cannot fit through the birth canal because the pelvis is too small, the baby is too big or if there is a mal presentation. If the woman in labor does not die, the pressure of the baby's head on the mother's pelvis leads to the death of tissue in the birth canal which creates a hole called an obstetric fistula. From this hole, urine or faeces constantly leak. The majority of women also deliver a stillborn baby. Fistula is completely preventable if obstructed labor is diagnosed early and if appropriate timely intervention occurs, which often includes the performance of a caesarean section18. Women suffering from fistula live with chronic urinary and fecal incontinence, the social effects of which include divorce, abandonment and abuse. Many women report feeling ashamed about their condition and therefore alienate themselves from friends and family^1^.

WHO has described vaginal fistulas as “the single most dramatic aftermath of neglected childbirth”. Vaginal fistulas are widespread in developing nations, mainly in sub-Saharan African and South Asian countries, where the social culture encourages marriage at a young age, of ten shortly after the girls' first menstrual period between the ages of 9 to 15 13. In many of these cases the first pregnancy is following soon after marriage^6^. Prior to mature age, the pelvis of a woman is not fully developed and chronic malnutrition can also further constraint its dimensions.

Women affected by obstetric fistula are often abandoned by their husbands, stigmatized by the community, physically debilitated and even blamed for their condition. Social isolation and abandonment often lead to low self-esteem, depression and prolonged emotional trauma^19^.

Information from various literature shows that obstetric fistula appears to be linked to certain social-economic and cultural factors including young age at marriage, poverty and illiteracy, living in rural areas with lack of emergence obstetric care^4^,^5^,^12^,^19^. Obstetric fistula has serious social and economic consequences on the lives of these women. Majority of the women are abandoned by their spouses who cannot stand the smell of urine. Major risk factors for obstetrics fistula include early age at pregnancy, short stature, illiteracy, poverty, not attending antenatal care, and rural place of residence or living far away from a health facility^14^.

Tesfaye^17^ used the Cox proportional hazard analysis to evaluate time to recovery of obstetric fistula at Yirgalem Fistula Hospital in Ethiopia and found that older ages at first marriage, weight less than 50 kg, height greater than 150cm, follow up of antenatal care, delivery at health center, duration of labor for less than 2 day, vaginal delivery, length and width of fistula less than 5cm and intact urethra significantly contribute to shorter stay in hospital to be treated and become physically cured.

Obstetric fistula remains a major public health problem in developing world where unattended obstructed labor is common and maternal mortality is unacceptably high. It is a tragedy in developing world because of illiteracy, poverty, ignorance and lack of health facilities^2^. An obstetric fistula is preventable and treatable condition, the untreated condition remains in developing countries. Ethiopia is one example of developing countries with poor maternal health care as well as high prevalence of obstetric fistula^20^. In Ethiopia approximately 26,000 women living with this disability with an additional 9000 new cases annually^11^. Typical fistula patients in Ethiopia are young peasant girls who are married in their early teens to farmers with little or no education. The girls are given heavy tasks in the household and are poorly educated. They have no access to any health institution during pregnancy and in labor are often helped during labor by women of the village to deliver at home and usually deliver a dead baby after being in labor for days^10^.

Many research findings have documented about the most important immediate clinical causes of obstetric fistula. But in Ethiopia, the underlying factors and consequences of the problem are not yet fully identified and adequately documented. Understanding the epidemiology of obstetric fistula and its determinants helps to design appropriate interventions on the basis of scientific evidences. As a result, this study tries to identify the risk factors asso ciated with determinants of obstetric fistula in Ethiopia using multiple logistic models.

## Objectives of the study

The general objective of this study was to examine the determinant factors associated with the prevalence of obstetric fistula in Ethiopia.

## Specific Objectives

- To assess the effect of socio-economic, demographic, environmental and health related factors associated with the occurrence of obstetric fistula.
- To determine the prevalence of obstetric fistula in Ethiopia.

## Methods and materials

### Data Source and sampling procedure

The data source for this study was the Ethiopian Demographic and Health Survey (EDHS) conducted by central Statistical Agency (CSA) in 2005. It is the second survey conducted in Ethiopia as part of the world wide Demographic and Health Survey Project. The survey was primarily designed to collect data on fertility, family planning, maternal care, infant and child mortality, childhood illnesses, malaria, nutrition, prevalence of female genital cutting, prevalence of obstetric fistula, knowledge of AIDS and other sexually transmitted infections in Ethiopia. The 2005 Ethiopia Demographic and Health Survey was designed to provide estimates for the health and demographic variables of interest for the following domains: Ethiopia as a whole; urban and rural areas (each as a separate domain); and 11 geographic regions (9 regions and 2 city administrations).

The 2005 EDHS is a nationally representative survey and of individual women were interviewed face to face on their background characteristics as well as reproductive health issues. The survey was carried out in two stages. In the first stage, 540 clusters (145 urban and 395 rural) were selected from a list of enumeration areas from the 1994 Population Census. In the second stage, a complete listing of households was carried out in each selected cluster. The 2005 EDHS collected a complete household listing which was prepared for each selected cluster and households. Households were systematically selected from each cluster for participation in the survey. In the survey, women were asked whether they have ever experienced obstetric fistula (OF) in their life. Only 3,178 of them responded about their experience on OF which was be considered in this study.

### Variables in the study

#### Dependent variable

The response variable for the individual is represented by and it measures women's experience of obstetric fistula and it is dichotomized with 1 being experienced and 0 not experienced.

#### Independent variables

Predictor variables are those variables which are presumed to affect or determine a dependent variable. Since based on the reviewed literature, some of the common predictors that are expected to influence determinants of obstetric fistula in Ethiopia were recorded as given below for the purpose of the analysis. In this study possible determinants of obstetric fistula were grouped as demographic, socio-economic, environmental and health related factors.

**i)** *Demographic related factors*

In this study the independent variables such as age at first marriage, age at first birth and marital status are expected to demographic risk factors.

**ii)** *Socio-Economic related factors*

In this study educational status, employment status and wealth index are included in socio-economic factors.

**iii)** *Environmental and health related factors*

Environmental and health related factors which will be included in this study are region, place of residence, place of delivery, body mass index and frequency of antenatal visits.

#### Statistical data analysis

The purpose of this study was to analyze the impact of women's demographic, socio-economic and environmental and health related factors that determine obstetric fistula in Ethiopia. The analysis was carried out in two parts. In the first part, results of descriptive statistics are presented; in the second part, we identified and examined determinants of obstetric fistula using multiple logistic regression analysis with the help of SPSS software.

#### Results of descriptive statistics

A total of 3178 women were included in the study from EDHS 2005 sample. The initial population which consisted of 14,070 women all were interviewed face to face on their background characteristics as well as reproductive health issues. Out of which, 3178 women had complete measurements and were considered in this study and others were excluded due to incompleteness of data on the variables which were considered in the analysis. From the sampled data, the prevalence of obstetric fistula was about 18.8% in Ethiopia.

The major socio-economic and demographic background characteristics of the respondents are presented in Table 1. Among 3178 respondents 84.6% were residents of rural areas and 15.4% are resides in urban areas. The higher prevalence of obstetric fistula occurred for women resides in rural areas (21.2%) as compared to women in urban area (5.4%).

**Table 1:** Distribution of Socio-economic and demographic related determinant factors of obstetric fistula in Ethiopia.

Variables	Categories	Counts (%)	Being Experienced OF		d.f	Chi-Square	P-Value
			No	Yes			
Age at first Marriage	Below 15 years	1022(32.1)	78.5%	21.5%	3	18.542	0.000*
	15 – 19 years	1626(51.2)	84.4%	16.6%			
	20 – 24 years	439(13.8)	81.1%	18.9%			
	25 years and above	91(2.9)	74.3%	25.7%			
Age at first Birth	Below 15 years	254(8.0)	70.1%	29.9%	3	10.767	0.013*
	15 – 19 years	1820(57.3)	80.6%	19.4%			
	20 – 24 years	886(27.9)	84.5%	15.5%			
	25 years and above	218(6.8)	86.2%	13.8%			
Educational Status	No education	2391(75.2)	80.6%	19.4%	2	253.41	0.000*
	Primary	524(16.5)	83.8%	16.2%			
	Secondary and Higher	263(8.3)	82.1%	17.9%			
Place of Residence	Urban	491(15.4)	94.6%	5.4%	1	176.77	0.000*
	Rural	2687(84.6)	78.8%	21.2%			
Marital Status	Married	2998(94.3)	81.1%	18.9%	2	2.493	0.288
	Widowed	74(2.3)	79.7%	20.3%			
	Divorced	106(3.4)	86.8%	13.2%			
Wealth Index	Poor	1412(44.4)	86.8%	13.2%	2	74.301	0.000*
	Middle	562(17.7)	83.6%	16.4%			
	Rich	1204(37.9)	73.7%	26.3%			
Employment Status	Currently working	2165(68.1)	84.0%	16.0%	1	33.092	0.000*
	No currently working	1013(31.9)	75.3%	24.7%			

* significant at 5%

Table 1 also shows that among the total respondents, 31.9% of them had no work and a higher prevalence of obstetric fistula was observed (24.7%). Majority of women (51.2%) were in their first marriage at an the age ranging between 15–19 years, while 32.1% of women's first marriage at in the age range below 15 years, about 13.8 % of women were first married at an age ranging between 20–24 years and the remaining 2.9% of women were first marriaed at an age of 25 years and above. The highest prevalence of obstetric fistula was observed for women whose first marriage was at an age range 25 years and above (25.7%) followed by women's whose first marriage at in the age range below 15 years (21.5%).

According to Table 1, age at first birth was found to be an important determinant factor of obstetric fistula. The proportion of women suffered from obstetric fistula is highest among teenage women's, age at first birth below 15 years (29.9%). The proportion of women suffered from obstetric fistula is also considerably higher for women first birth at in the age range between 15–19 years (19.4%) compared to those whose first birth was between the age range 20–24 years (15.5 %) and women's first birth at an the age range of 25 years and above (13.8 %).

Furthermore, Table 1 shows that, the proportions of women who suffered from obstetric fistula varied with educational status. Majority of respondents 75.2% of them had no education. While, only 16.5% and 8.3% of them had primary education level and secondary and higher education level respectively. The highest prevalence was observed for women who had no education (19.4%).

Among the socio-economic and demographic determinant factors, age at first marriage, age at first birth, educational status, wealth index and employment status were found to have a significant effect on the incidence of obstetric fistula at 5% levels of significance.

The major environmental and health related background characteristics of the respondents are presented in Table 2. The proportion of women who suffered from obstetric fistula varied from one region to another. The highest prevalence of obstetric fistula was recorded in Amahara (30.8%) followed by Oromia (27.4%) and Gambella (24.1%) as opposed to lowest prevalence which was recorded in Addis Ababa (6.3%) followed by Benshangul Gumuz (6.4%).

**Table 2:** Distribution of environmental and health related determinant factors of obstetric fistula in Ethiopia

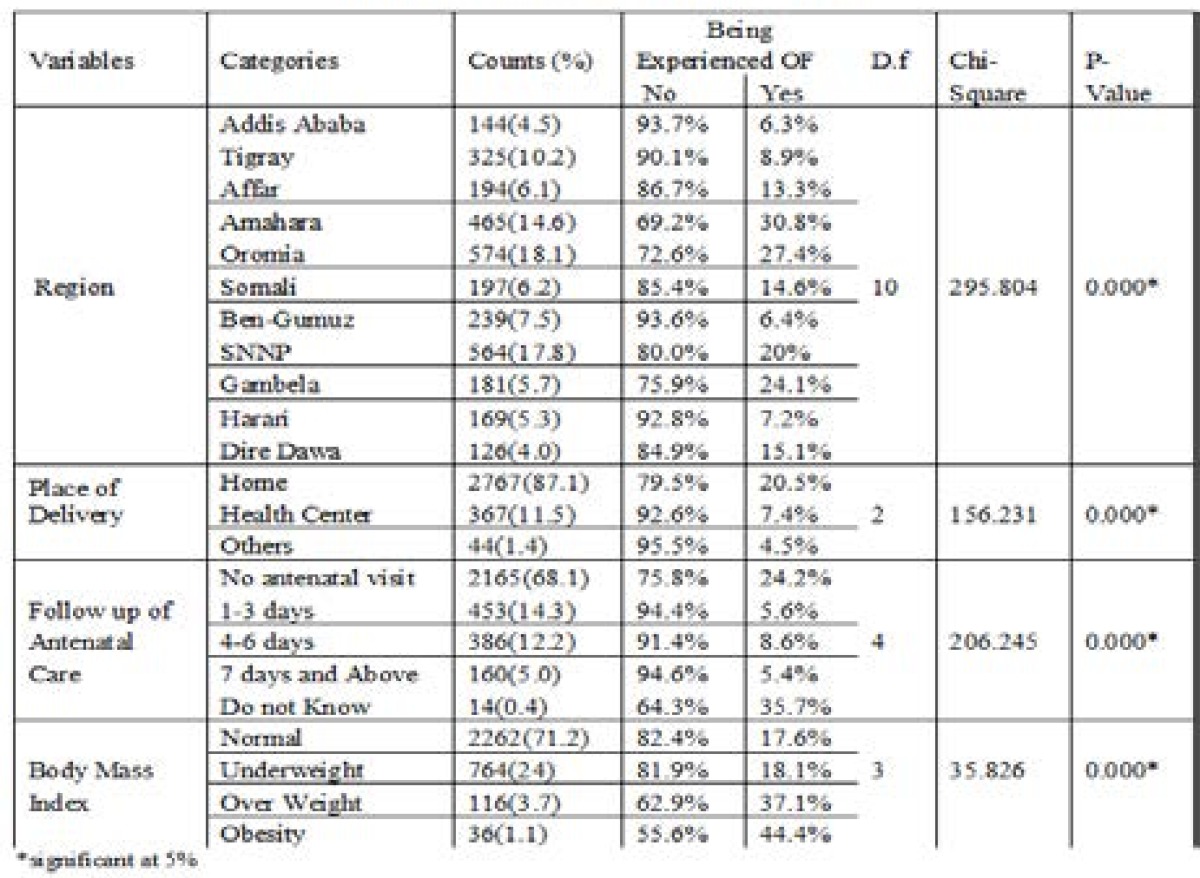

Table 2 also shows that there is a significant association between incidence of obstetric fistula and place of delivery (p<0.001). Surprisingly, among the whole respondents about 87.1% of them are delivered at their home and the highest prevalence of fistulas was recorded in these (20.5%) compared to women were delivered at health center (7.4%) followed by women were delivered at another place (4.5%). This showed that delivering at health center would help to decrease the number of patients exposed to obstetric fistula. Moreover, results presented in Table 2 show that antenatal care visits and body mass index are important variables. The highest proportion of women who suffered from obstetric fistula was observed among obese women that means BMI >30 (44.4%) followed by overweight (BMI between 25 and 29.9 (37.1%)) as opposed to the lowest proportion which was recorded in women who have normal weight (BMI between 18.5 and 24.9) and followed by underweight (BMI<18.5). Similarly, the highest proportions of obstetric fistula were observed among women who did not know about antenatal visits (35.7%) and had no antenatal visits (24.2%) as compared to women's who had taken antenatal care for one and more days during pregnancy.

#### Binary logistic regression analysis

Binary logistic regressions were fitted based on chi-square test result of bivariate analysis. Based on results presented in Table 1 and 2, those predictor variables that are associated with obstetric fistula at 5% level of significance were selected for multiple logistic regression analysis. Multiple logistic regression models were fitted using these predictor variables using forward selection (Likelihood ratio) method. The results are presented in Table 3 showed that eight of the predictor variables were significantly associated with the incidence of obstetric fistula.

**Table 3:** Model Summary of Binary Logistic Regression

−2 Log likelihood	Cox & Snell R Square	Nagelkerke R Square
2552.525^a^	0.15	0.242

#### Assessment of Goodness of Fit of the Model

For categorical data, after a logistic regression model has been fitted, a global test of goodness of fit of the resulting model should be performed. It is necessary to see the appropriateness, adequacy and usefulness of the fitted model. The most commonly used techniques are Likelihood-Ratio test, Hosmer and Lemeshow test, R-Square and the Wald goodness of fit test.

#### Likelihood-Ratio Test

The most common assessment of overall model fit in logistic regression is the likelihood Ratio test, which is the chi-square difference between the null model with the constant only and the model containing a set of predictors. Under model summary in Table 4.3, we see that −2Log Likelihood statistics is 2552.525. This statistics show us how much improvement is needed before predictors provide the best possible prediction of the response variable, the smaller the statistics the better the model. The statistics for only intercept model is =. The inclusion of the parameters reduced the statistics by, which is reflected chi-square for omnibus test. The result (, p-value<0.001), shows that the model is adequate, meaning that at least one of the predictors is significantly related to the dependent variable. That is, the null hypothesis is that there is no difference between the model with only a constant and the model with independent variables was rejected. The Hosmer and Lemeshow goodness of fit test divides subjects into deciles based on predicted probabilities, then computes a chi-square from observed and expected frequencies in a 102 table. A non-significant chi-square indicates that there is no difference between the observed and the model predicted values and hence estimates of the model adequately fit the data. Since Table 4 shows as the p-value is 0.844 and it is greater than 0.05 then, we don't reject the null hypothesis that there is no difference between observed and model predicted values, implying that the model fitted the data well.

**Table 4:** Hosmer and Lemeshow Test

Chi-square	Df	Sig.
4.139	8	0.844

#### Results of logistic regression analysis

Logistic regressions were used to analyze the effect of each independent variable on women's status of obstetric fistula, while controlling for the other independent variables. Accordingly region, place of residence, educational status, age at first birth, age at first marriage, employment status, place of delivery and follow up of antenatal care were found to be significant predictors for prevalence of obstetric fistula at 5% level of significance (see Table 4.5). Thus, the estimated model is given by:

Where: Predicted probability of obstetric fistula, constant, place of residence of women at level 1, women's region level of educational background of women, age at first marriage of women's at level, age at first birth of women's at level, employment status of women's at level 1, place of delivery of level, follow up of antenatal care of level.

Therefore, based on the result of Table 5, the final logistic regression equation consisting of the significant variables is given by:

$$\begin{array}{l} \begin{array}{ll} \text{logit(}\pi \text{(X)) =} & 2.742+0.254{\text{Reg}}_{\text{1}}+0.95{\text{Reg}}_{\text{2}}+1.536{\text{Reg}}_{\text{3}}+\cdots +0.669{\text{Reg}}_{\text{10}}+1.642{\text{PIR}}_{\text{1}}\\ & -2.232{\text{EduSta}}_{\text{1}}-1.609{\text{EduSta}}_{\text{2}}-2.068{\text{Ag1Ma}}_{\text{1}}-1.696{\text{Ag1Ma}}_{\text{2}}-0.416{\text{Ag1Ma}}_{\text{3}} \end{array}\\ \begin{array}{ll} \; & -0.733{\text{Ag1Bi}}_{\text{1}}-1.719{\text{Ag1Bi}}_{\text{2}}-2.031{\text{Ag1Bi}}_{\text{3}}+0.284{\text{EmSt}}_{\text{1}}-1.570{\text{FolAnt}}_{\text{1}}\\ & \text{+}\cdots -0.945{\text{FolAnt}}_{\text{4}}-1.625{\text{PlDel}}_{\text{1}}-0.878{\text{PlDel}}_{\text{2}} \end{array} \end{array}$$

**Table 5:** Maximum likelihood estimates of predicting the incidence of Obstetric fistula in Ethiopia.

	Categories	B(S.E.)	Wald	df	Sig.	Exp(B)	95.0% C.I. for EXP(B)	

							Lower	Upper
Region			134.518	10	.000*			
	Addis Ababa(Ref)							
	Tigray	0.254(0.301)	0.712	1	.398	1.289	0.715	2.326
	Affar	0.950(0.331)	8.237	1	.004*	2.585	1.351	4.947
	Amahara	1.536(0.323)	22.614	1	.000*	4.646	2.467	8.750
	Oromia	1.483(0.338)	19.227	1	.000*	4.405	2.270	8.547
	Somali	1.074(0.314)	11.681	1	.001*	2.928	1.581	5.423
	Ben-Gumuz	0.122(0.305)	0.160	1	.689	1.130	0.621	2.054
	SNNP	1.278(0.308)	17.217	1	.000*	3.589	1.963	6.565
	Gambella	1.370(0.313)	19.087	1	.000*	3.934	2.128	7.272
	Harari	0.240(0.317)	0.573	1	.449	1.271	0.683	2.366
	Dire Dawa	0.669(0.321)	4.337	1	.037*	1.952	1.040	3.664

Place of Residence	Urban (Ref) Rural	1.642(0.190)	74.908	1	.000*	5.167	3.562	7.495

Educational			96.086	2	.000*			
Status	No education(Ref)							
	Primary	−2.232(0.235)	89.994	1	.000*	0.107	0.068	0.170
	Secondary & Higher	−1.609(0.246)	42.686	1	.000*	0.200	0.123	0.324

Age at first			58.369	3	.000*			
Marriage	<15 years(Ref)							
	15–19 years	−2.068(0.390)	28.161	1	.000*	0.126	0.059	0.271
	20–24 years	−1.696(0.376)	20.356	1	.000*	0.183	0.088	0.383
	25 and above years	−0.416(0.376)	1.226	1	.268	0.660	0.316	1.378

Age at first			96.663	3	.000*			
Birth	<15 years(Ref)							
	15–19 years	−0.733(0.243)	9.082	1	.003*	0.481	0.298	0.774
	20–24 years	−1.719(0.195)	78.13	1	.000*	0.179	0.122	0.262
	25 and above years	−2.031(0.336)	36.619	1	.000*	0.131	0.068	0.253

Employment	Currently working (Ref)							
Status	Not currently working	0.284(0.114)	6.218	1	.013*	1.328	1.063	1.660

Follow up			82.661	4	.000*			
of Antenatal	No antenatal visit(Ref)							
Care	1–3 days	−1.570(0.233)	45.464	1	.000*	0.208	0.132	0.328
	4–6 days	−1.526(0.245)	38.656	1	.000*	0.217	0.134	0.352
	7 days and Above	−2.050(0.448)	20.959	1	.000*	0.129	0.053	0.310
	Do not Know	−0.945(0.894)	1.118	1	.290	0.389	0.067	2.241

Place of			26.733	2	.000*			
Delivery	Home(Ref)							
	Health Center	−1.625(0.325)	24.96	1	.000*	0.197	0.104	0.373
	Others	−0.878(0.564)	2.423	1	.120	0.416	0.138	1.256

	Constant	2.742(0.526)	27.209	1	.000*	15.512		

* Significant at 5%, Ref = reference category, OR=Odd ratio estimate

The logistic model showed that the likelihood of having obstetric fistula was significantly significant with geographical regions. Women who were living in Amhara region were 4.646 times more likely to have experience dof obstetric fistula than Addis Ababa region controlling for other variables in the model (OR=4.646; 95% CI: 2.467 8.750). Similarly, Women who lived in Oromia region were 4.405 times more likely to have experienced obstetric fistulas than Addis Ababa controlling for other variables in the model (OR=4.405; 95% CI: 2.270–8.547). Moreover, women who live in Affar, Somali, SNNP, Gambella and Dire Dawa were more likely to have experienced obstetric fistula than Addis Ababa region. Unlikely the odds of having obstetric fistula among women who live in Tigray, Benshangul gumuz and Harari were not significantly differ from that of women who live in Addis Ababa region. Table 5 also shows that place of residence has a significance effect with the incidence of obstetric fistula. A woman who resided in a rural area was 5.167 times more likely to have obstetric fistula than that of woman who resided in urban area controlling for other variables in the model (OR=5.167; 95% CI: 3.562–7.495).

The logistic model showed that women's educational status has a negative effect on the incidence of obstetric fistula. A woman having primary education was 89.3% less likely to have obstetric fistula than women who had no education (OR=0.107; 95% CI: 0.068–0.17). Similarly, Women having secondary and higher education were 80% less likely to suffer obstetric fistula than women who had no education controlling for other variables in the model (OR=0.200; 95% CI: 0.123–0.324).

According to result Table 5, we observe that the log of the odds of women who suffered from obstetric fistulas were negatively related to age at first marriage. Women whose first marriage was at an age ranging between 15–19 years were 87.4% less likely to suffer obstetric fistula than women whose first marriage was at an age ranging <15 years. In the same way, women whose first marriage was at an age ranging between 20–24 years were 81.7% less likely to suffer obstetric fistula than women whose first marriage was at an age <15 years controlling for other variables in the model. Similarly, the logistic model showed that age at first birth also had a negative significant association with the incidence of obstetric fistula (p<0.001).

The logistic model showed that women's employment status is a significant predictor of the incidence of obstetric fistula. Women who were not currently working were 1.328 times more likely to have experienced of obstetric fistula than women who had currently working controlling for other variables in the model (OR=1.328; 95% CI: 1.063–1.660). The analysis also showed frequency of antenatal care visits has a statistically significant association with the incidence of obstetric fistula (p<0.001). The odds of women experiencing obstetric fistula among those who had taken antenatal care visits for 7 days and above during pregnancy was 87.1% less likely to occur compared to women who had no antenatal care visit (OR=0.129; CI: 0.053–0.310). Similarly, the odds of women experiencing obstetric fistula among those who had taken antenatal care visits for 1–3 days during pregnancy was 79.2% less likely to occur compared to women who had no antenatal care visit controlling for other variables in the model (OR=0.208; 95% CI: 0.132,0.328). Furthermore, place of delivery had a significant effect with the incidence of obstetric fistula. Women who delivered from health centers were 80.3% less likely to suffer from obstetric fistula compared to women who delivered from their homes controlling for other variables in the model (OR=0.197; CI: 0.104–0.373).

## Conclusion and recommendations

The study identified that demographic, socio-economic, environmental and health related variables have an important effect on determinants of obstetric fistula in Ethiopia. The results which are obtained are discussed as follow:

The descriptive analysis of this study showed that the prevalence of obstetric fistula in Ethiopia was 18.8%. Based on the result of this study, woman who live in Amhara, Oromia, Gambella, SNNP, Somali, Affar and Diredawa regions were more likely to have experiencing obstetric fistula than women who live in Addis Ababa region.

This study found that experiencing of obstetric fistula was significantly associated with age at first birth. Women whose first birth was at an age ranging between 15–19 years were 51.9% less likely to suffer obstetric fistula than women whose first birth was at an age of <15 years. On the same way, women whose first birth was at an age range 25 years and above were 86.9% less likely to suffer obstetric fistula than women whose first birth was at an age range of <15 years. This result is in agreement with Muleta^11^ and Roka et al^16^, revealed that early age at pregnancy has one of the factors leading to increase risks of obstetric fistula with particular reference to adolescent's women (12–19 years). This finding showed that there is an inverse relationship between age at first birth and prevalence of fistula.

The finding also showed that place of delivery and follow up of antenatal care were found to be statistically significant with the incidence of obstetric fistula. Women who delivered from a health facility and followed antenatal care for more than one day were less likely to exposed obstetric fistula than those women who delivered from their homes and had no antenatal care visits. This result is in agreement with Roka et al.^16^ who suggested that major risk factors for obstetric fistula were not attending antenatal care and living far away from health facility. Similarly, the finding is consistent with Muleta11 who found that women had little or no access to healthcare, prenatal or emergency obstetric care were the most frequently cited problems suffered to obstetric fistula. Moreover, the result is also correspondence with Mohamed et al^10^ revealed that the victim of obstetric fistula was mostly not attending on regular antenatal care and most deliveries were carried at home, attended by traditional birth attendants.

The result of this study indicated that incidence of obstetric fistula was significantly associated with educational status. Women who had primary education, secondary and higher education were less likely to suffer obstetric fistula than illiterate women. This result is consistent with Roka et al.^16^ Wall^21^ and Yeakey^23^ found that the major risk factors for obstetric fistula were illiteracy. Similarly, the finding is correspondence with Tebekew^17^ who showed that women with secondary and higher education were 78 % less likely to affect obstetric fistula than those who had no education. Furthermore, the result is also in agreement with Mohamed et al.^10^ revealed that the victim of obstetric fistula was mostly illiterate. Another study done by Tebeu^18^ gives a general conclusion to this important factor; education plays an important role in the occurrence of obstetric fistula, and in maternal mortality and morbidity.

In this study, place of residence was a major cause problem of obstetric fistula, especially in developing countries like Ethiopia. The study showed that the likelihood of women who reside in rural areas to suffer obstetric fistulas was 5.167 times more than those women who reside in urban areas. Most literature reviewed about this important determinant factor of obstetric fistula, for instance a study done by Wall 21; Holme et al 4 and Nathan^14^ showed that the major risk factors for obstetric fistula were rural place of residence. This finding also corresponds to a study done in Ethiopia that revealed that majority of rural women were affected by obstetric fistula, Tebekew^17^.

The model of this study revealed that likelihood of having obstetric fistula among women who had no current employment was 1.328 times more likely to have experience of obstetric fistula than women's who had currently employed. This finding is consistent with a study done in West Pokot by Mabeya 7 revealed that the majority of fistula incidents occurred in women had no specific occupation.

Finally, according to the result of this study, multiple logistic regression showed that region, place of residence, educational status, age at first marriage, age at first birth, employment status, place of delivery and follow up of antenatal care were all important factors to determining the incidence of obstetric fistula in Ethiopia.

## Recommendations.

Sudanese journal of public health, 4(2).

Based on the findings of this study, the following recommendations are forwarded:

- To avoid the risk of obstetric fistula due to pregnancy and delivery, strengthening family planning and antenatal care services should be addressed extensively.
- Awareness has to be given for the society on the risk of early marriage and early pregnancy. So that, the Ethiopian government should ensure the practice of the law against early marriage through enhancing family and community awareness about the dangers of early marriage and early pregnancy.
- On the basis of the study findings, improving the educational and employment status of women with the aim of enhancing their socio-economic status is vital for their health wellbeing.

## References

[1] Barone Mark (2010). Determinants of post-operative outcomes in fistula repair surgery.

[2] Dangal G, Thapa K, Yangzom K, Karki A (2013). Obstetric Fistula in the Developing World: An Agonising Tragedy. NJOG.

[3] EDHS (2005). Ethiopian Demographic and Health Survey.

[4] Holme A, Breen M, Mac Arthur C (2007). Obstetric fistula a study of women managed at the Monze Mission Hospital, Zambia. BJOG.

[5] Johnson K (2007). Incontinence in Malawi: Analysis of a proxy measure of vesico vaginal fistula in a national survey. International Journal of Gynaecology and Obstetrics.

[6] Karen M (2009). Social Implications of Obstetric Fistula: an Integrative Review. Journal of Midwifery & Women's Health.

[7] Mabeya HM (2003). Characteristics of women admitted with obstetric fistula in the rural hospitals in West Pokot, Kenya.

[8] Ministry of Health of Federal Democratic Republic of Ethiopia (2003). Health and healthrelated indicators.

[9] MOH (2007). Health and Health Related Indicators.

[10] Mohamed Y, Boctor A, Ahmed A, Seedahmed A, Abdelgadir A, Abdalla M (2008). Contributing factors of vesico-vaginal fistula (VVF) among fistula patients in Dr. Abbo's National Fistula & Urogynecology Centre in Khartoum. Sudanese Journal of Public Health.

[11] Muleta M (2004). Socio-Demographic Profile and Obstetric Experience of Fistula Patients Managed at the Addis Ababa Fistula Hospital. Ethiopian Medical Journal.

[12] Muleta M (2006). Obstetric Fistula in Developing Countries. J Obstet Gynaecol.

[13] Muleta M, Hamlin C, Fantahun M, Kennedy R, Tafesse B (2008). Health and Social Problems Encoun tered by Treated and Untreated Obstetric Fistula Patients in Rural Ethiopia, Addis Ababa Fistula Hospital, Addis Ababa, Ethiopia. J Obstet Gynaecol.

[14] Nathan LM, Rochat HC, Bank E, Gringorescu B (2008). Obstetric fistula in West Africa: patients' perspectives. Am J Obstet Gynecol.

[15] Narcisi L (2010). The Fistula Crisis in Sub-Saharan Africa: an Ongoing Struggle in Education and Awareness. Urologic Nursing.

[16] Roka G, Akech M, Wanzala P, Omolo J, Gitta S, Waiswa P (2013). Factors associated with obstetric fistula occurrence among patients attending selected hospitals in Kenya.

[17] Tebekaw Y (2011). Obstructed social services leading to obstetric fistula in Ethiopia: Evidence from DHS data.

[18] Tebeu PM (2009). Risk factors for obstetric fistula: a clinical review. International Urogynecology Journal.

[19] Tesfaye G (2013). Survival Analysis of Time to Recovery from Obstetric Fistula at Yirgalem Hamlin Fistula Hospital, SNNPR, Ethiopia.

[20] Wall (2012a). A Framework for Analyzing the Determinants of Obstetric Fistula Formation. From Studies in Family Planning.

[21] Wall LL (2006). Obstetric Vesico vaginal Fistula as an International Public-Health Problem. the Lancet.

[22] WHO (2005). Obstetric fistula, Guiding Principles for Clinical Management and Program Development.

[23] Yeakey M (2009). The lived experience of Malawian women with obstetric fistula. Culture, Health & 38; Sexuality.

